# From dyadic deco-construction to reco-construction: a framework for the human–osteoarthritic dog relationship

**DOI:** 10.3389/fvets.2026.1828548

**Published:** 2026-08-12

**Authors:** Laurie Martin, Colombe Otis, Bertrand Lussier, Eric Troncy

**Affiliations:** Groupe de Recherche en Pharmacologie Animale du Québec (GREPAQ), Université de Montréal, Saint-Hyacinthe, QC, Canada

**Keywords:** canine, chronic, detection, diagnostic, management, owner, pain, self-determination

## Abstract

Chronic pain (CP) associated with canine osteoarthritis (OA), remains under-detected, under-diagnosed, and insufficiently managed, while the early signals prompting owners to consult a veterinarian are poorly characterized. This narrative review draws on self-determination theory (SDT) to explore how the experience of OA-associated CP reshapes the self-determination capacities within owner–dog dyads. It describes the deco-construction of dyadic co-construction induced by the apparition and progression of OA-associated CP, followed by its reco-construction after the veterinary diagnosis, integrating a pivotal moment: the owner’s rupture point. Its objectives are twofold: (1) from a SDT perspective, to conceptualize these processes when the dog develops OA-associated CP; and (2) to propose strategies that help dyads navigate them more effectively and trigger the rupture point earlier. Deco-construction progressively erodes dyadic self-determination. While the accumulation of CP-related signals may lead the owner to their rupture point, their perceived competence, denial, wait-and-see attitude and apprehension of veterinary cost often delay it. Reco-construction relies on respecting the self-determination of the dog, the owner, and the veterinarian. At home, the adaptation of therapeutic strategies enables the dyad to regain a sense of mutual self-determination. These processes are influenced by biopsychosocial, experiential, environmental, and technical factors specific to the dog, the human, and the disease. A strong co-construction appears to facilitate them. To better capture the dynamics of co-/deco-/reco-construction and anticipate owners’ rupture point, several tools appear promising, but none fully integrate co-construction, bidirectionality, and dyadic attachment. There is an urgent need to develop more integrative conceptual frameworks and tools. This review remains conceptual and narrative in nature (albeit based on an exhaustive literature), and the proposed framework has not yet been clinically validated.

## 1 Introduction

In a recent narrative review, the co-construction of owner/dog (dyadic) relationships was conceptualized through the lens of the self-determination theory (SDT) (1). The present review builds upon this foundation and extends the theoretical framework to include the concepts of deco-construction and reco-construction in dyads facing canine osteoarthritis (OA) chronic pain (CP).

Originally, SDT proposes that human well-being depends on the ability to satisfy basic psychological needs for motivational purposes—in other words, to be self-determined (2). Motivation is situated along a continuum ranging from intrinsic motivation, driven by pleasure and interest, to amotivation, characterized by disengagement (2). Between these two poles lies extrinsic motivation, which is more instrumental in nature (2). These motivational orientations are linked to individual capacities for satisfying the needs for autonomy (a sense of control over one’s actions), competence (a sense of effectiveness), and relatedness or attachment (a sense of connection and social support) (2). The dyadic owner/dog co-construction can therefore be described as an adaptive, dynamic, and evolving process that aims to create and maintain the relationship (1). It is influenced by biopsychosocial, experiential, and environmental factors specific to each partner, whose interaction gives rise to a unique dyad with distinctive capacities for self-determination satisfaction (1).

The co-construction may be deco-constructed by factors affecting the dog, the human, or the dyad itself. Dyadic deco-construction is defined as a progressive disruption in interactions (e.g., functional, emotional, behavioral) and motivations between a dog and its caregiver. This process may lead to a reduced reciprocity, relational predictability, and fulfillment of dyadic basic psychological needs. Canine CP is one of the factors of dyadic deco-construction and represents a major challenge: it is pain that persists beyond the expected period of tissue healing or could occur in absence of ongoing injury (3). CP is a multidimensional experience encompassing sensory-discriminative, cognitive, emotional, and behavioral components (4), which requires long-term, often multimodal, management (5, 6). As soon as diagnosis and management plan are actualized, a dyadic reco-construction could begin. It is defined as an adaptive and evolving reorganization of dyadic interactions and motivations. It aims to (partially) restore and maintain affected relational reciprocity, predictability of interactions, and satisfaction of the dyad’s psychological needs. Reco-construction may include behavioral, environmental, therapeutic, and relational adjustments allowing the dyad to develop new coping strategies and support its updated self-determination despite the CP presence. SDT-based approaches in human healthcare provide a relevant perspective for understanding this experience, as they explore how motivation and basic psychological need(s) satisfaction shape the well-being of both human patients and caregivers (2, 7–9). For example:

- A systematic review (*N* = 5) with meta-analysis showed that exercise programs incorporating SDT had a significant positive effect on disability and quality of life in patients with chronic low back pain (7).
- An integrative review (*N* = 10) revealed that the well-being of informal caregivers of patients with long-term illness appears to be closely tied to their autonomous motivation to provide care (8).

Although based on a limited number of studies, these findings suggest that improving access to self-determination for both human patients and informal caregivers throughout the experience of CP can enhance their overall well-being (7, 8). SDT provides a useful conceptual lens through which canine welfare and owner perceptions may be interpreted, although its application to non-human animals remains theoretical. By analogy, if an owner–dog dyad is built upon a favorable co-construction (1), *its reco-construction may similarly benefit from the principles of SDT*. Within this context, several challenges associated with the under-detection, under-diagnosis, and under-management of CP in animals could be re-examined:

1. *Expression of CP, emergence/perception of “abnormality” and decision to consult a veterinarian: deco-construction leading to the rupture point*. The initial expressions of canine CP are often subtle and intermittent before becoming permanent (e.g., increased withdrawal, reduced sociability/play, altered posture, gait changes, hesitation or refusal to perform certain activities, and overall reduction in activity compared to the dog’s normal baseline) (10). To gain better visibility, these manifestations could be understood as progressive alterations in the dog’s—and by extension, the dyad’s—ability to sustain self-determination. *Yet, this process has never been defined as a deco-construction nor analyzed through the lens of SDT*. The rupture point is conceptualized as a cognitive-behavioral threshold resulting from the accumulation of “abnormal” biopsychosocial, experiential, and environmental cues becoming salient enough to motivate the owner to seek professional veterinarian assistance (11). “Abnormality” includes both emergence of new, unusual behaviors, incompatible with normal aging, and disappearance of familiar behaviors. However, in the context of canine CP, it remains under-studied (11), under-conceptualized, and has never been approached from a SDT perspective.
2. *Diagnosis and management of CP: the reco-construction*. The diagnostic process relies on clinical history, physical examination, complementary tests, and negotiation between veterinarian and caregiver regarding the treatment plan (5). At home, subsequent readjustments may contribute to improving dyadic self-determination. *Yet, this adaptive process has never been defined as a reco-construction nor examined through SDT*.
3. Assessment tools for canine CP (Table 1). Several standardized questionnaires are available to assess canine OA CP (e.g., Helsinki chronic pain index [HCPI] (12), canine brief pain inventory [CBPI] (13), Liverpool osteoarthritis in dogs [LOAD] (14)). However, they remain underused (15), do not allow anticipation of the owner’s rupture point, and their relevance from a SDT perspective is incomplete. They rely on the owner’s autonomy and competence to accurately observe pain-related behaviors without directly addressing the canine perspective. The relational dimension is also overlooked. Yet, owners often struggle to recognize signs of CP in their dogs (11, 16, 17), and attachment may influence how they provide care (18–23).

**Table 1:** Comparison of the constructs measured by the main canine OA assessment and screening tools and by a potential rupture point screening tool (proposed framework) based on the concepts of co-construction, deco-construction, and reco-construction.

Analytical category	Assessment and screening tool				
	CBPI*	LOAD**	HCPI***	Canine OA**** screening checklist	Canine OA rupture point screening tool
Dyadic co-construction	No	No	No	No	Yes
Canine functional limitation	Yes	Yes	Yes	Yes	Yes
Canine psychological change	Low	Low	Partial	Partial	Yes
Canine behavioral change	Low	Low	Low	Yes	Yes
Canine pain severity	Yes	Partial	Partial	Partial	Partial
Owner cognitive and emotional processes	Low	Low	No	Partial	Yes
Anticipation of the rupture point	No	No	No	Partial	Yes

Dyadic co-construction refers to daily routines and the satisfaction of the basic psychological needs of the dog, the owner, and the dyad. Canine functional limitations correspond to a reduction in the dog’s ability to perform physical activities or daily-life tasks. Canine psychological changes include manifestations of anxiety, depression, prolonged sleep, or reduced sociability. Canine behavioral changes refer to observable modifications in usual behaviors, such as reluctance to perform certain activities or reduced mobility / emergence of abnormal behaviors. Canine pain severity corresponds to the intensity and perceived impact of pain. Owner cognitive and emotional processes encompass perceptions, beliefs, interpretations, emotions, and decision-making mechanisms related to the dog’s health condition. Finally, anticipation of the rupture point corresponds to the ability of a tool to suggest the screening of OA and to instill in the owner the need to consult a veterinarian. *Canine brief pain inventory, **Liverpool osteoarthritis in dogs, ***Helsinki chronic pain index, ****Osteoarthritis. “Low” means this category to partially derive from the functional alterations assessment.

**Figure 1:**
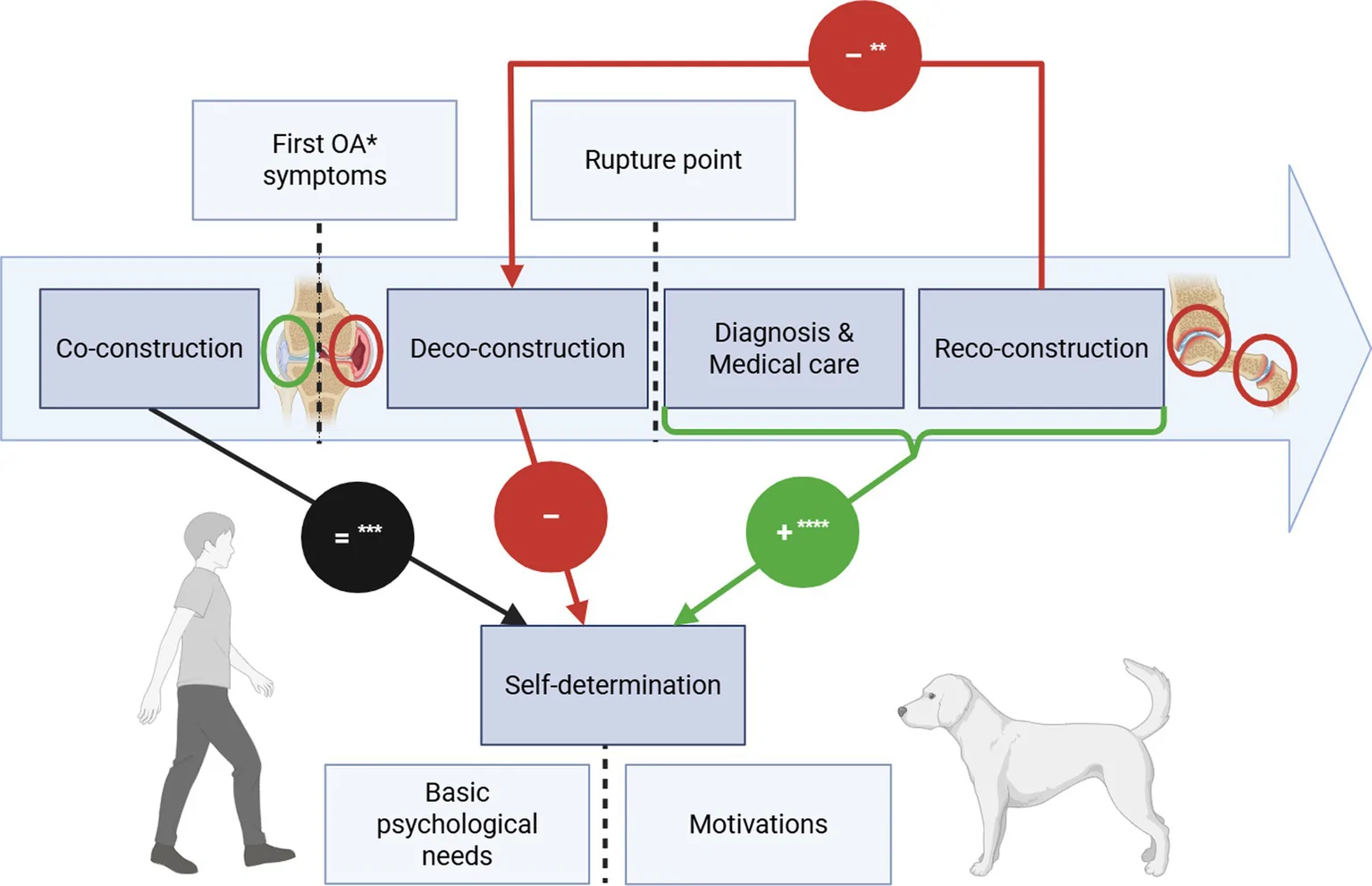
Dyadic co-construction affected by canine osteoarthritis, based on the self-determination theory. Osteoarthritis initiates a deco-construction characterized by emergent abnormalities within the dyadic co-construction. When cumulated chronic pain indicators reach significance for the caregiver, it may conduct to consulting a veterinarian (rupture point). Diagnosis and implementation of a therapeutic plan then initiate a dyadic reco-construction. Both processes are progressive, with functional, emotional, and behavioral interactions as well as motivations consequences. The lower part of the figure illustrates that dyadic self-determination influences, and may be influenced by, each of these processes. *Osteoarthritis; **Negative influence; ***Neutral influence; ****Positive influence. Created in BioRender. Martin (2026) https://BioRender.com/5i0jxp8.

These observations highlight the need to design tools capable of anticipating the owner’s rupture point and strengthening dyadic self-determination throughout the processes of deco-construction and reco-construction. The type of CP explored will be OA, a chronic, degenerative, and progressive disease of the entire joint organ, including tendons, muscles, and ligaments (24, 25). OA affects 10–20% of the adult canine population in Western countries (25). In the United Kingdom, it is considered the disease with the highest severity score and the second most detrimental to canine welfare (26). Yet, the challenges outlined above equally apply to OA (11, 25). These reflections lead to three hypotheses:

1. Canine OA CP induces a dyadic deco-construction. If the rupture point is reached and a diagnosis is made, a dyadic reco-construction may follow.
2. In the context of canine OA CP, SDT provides a relevant theoretical framework to conceptualize deco-construction, the rupture point, and reco-construction within the dyad.
3. Analyzing the interaction among dyadic co-, deco-, and reco-construction processes could help identify the owner’s initial rupture point and promote earlier detection and diagnosis of canine OA CP.

- The primary objective of this narrative review is to conceptualize, through SDT, the processes of dyadic deco-construction—including the owner’s rupture point—and reco-construction when a dog develops OA-associated CP (Figure 1). The secondary objective is to discuss potential solutions, and their limitations, that could help dyads navigate these processes more effectively and anticipate the rupture point. The processes discussed in this review were examined through the lens of canine SDT, with particular attention to the potential disruptions to self-determination capacities associated with canine OA-related CP. Throughout the manuscript, fundamental psychological needs and motivations, at the individual (canine and human) and dyadic levels, are used as a transversal interpretative framework to understand the different experiences described. SDT is therefore not applied to explain the biological mechanisms underlying OA, but rather to provide a conceptual framework for exploring how OA-associated CP may influence the self-determination capacities of the dog, the owner, and the human–dog dyad. For this reason, canine OA is briefly introduced before considering OA-associated CP, with particular emphasis on the recently recognized contribution of neuro-sensitization and nociplastic mechanisms. The underlying hypothesis is that these neurophysiological changes may contribute to behavioral modifications in dogs, which can be perceived by owners and may influence the interpretation of SDT-related constructs within the human–dog dyad.
- One pathway leading to the rupture point was selected, but other trajectories are possible. For example, at home, owners may overlook early signs of OA (11) and only become aware of the disease during a routine veterinary consultation. In this case, the veterinarian appears to initiate and guide the dyad toward its rupture point. Conversely, previous experience or better knowledge and skills within the dyad, whether related or unrelated to OA, may lead owners to request early screening before clinical signs become evident.
- Because the literature specifically addressing OA-associated CP in dogs remains limited for some of the topics discussed, evidence from studies investigating other chronic conditions was also considered when relevant. These studies were used to support the development of the proposed conceptual framework and to provide broader insights, rather than to suggest a direct equivalence between OA-associated CP and other disease states.
- The term dog owner was favored rather than a more inclusive terminology for companion animals (27). This choice avoids inferring owners’ perceptions about their dogs (28) as well as the quality of their dyadic co-construction (1).
- This work was not exhaustive and should be considered as a conceptual and exploratory contribution to a relatively underexplored field of study.

## 2 Dyadic deco-construction

### 2.1 OA and SDT

#### 2.1.1 OA, OA-associated CP and neuro-sensitization

Canine OA develops under the influence of multiple risk factors, including large body size, overweight and obesity, sterilization, previous joint injury and conformation defects (25). Genetic and breed predispositions are suspected but remain unconfirmed; age is a misleading indicator, as young dogs can be just as affected by this disease as older ones (25). Its cause may be indeterminate (e.g., conformation defects) or related to lifestyle factors (e.g., exercise, diet) or past injuries (e.g., dysplasia, fracture, cruciate ligament rupture) (29).

The progression of OA is described in four stages (30): from the first, preclinical stage—where the patient appears healthy but presents risk factors for developing the disease—to the fourth stage, where clinical signs are pronounced, persistent, and greatly affect the animal’s quality of life. Its canine impacts include (25, 31):

- *Pain* that fluctuates depending on the location, nature, and progression of the lesions and pathology.
- Associated alterations (linked to pain):
- *Orthopedic* (e.g., lameness, stiffness, changes in posture and gait).
- *Neurological* (e.g., neuro-sensitization, alteration in nervous conduction, or in sensorial perceptions).
- *Behavioral* (e.g., reluctance or refusal to perform certain activities, reduced mobility).
- *Psychological* (e.g., signs of depression, anxiety, prolonged sleep, decreased sociability).

These signs are initially subtle, intermittent, and insidious before becoming permanent in advanced stages (25). They do not manifest in the same way in all dogs. For example, a study involving *N* = 99 OA dogs suggested that only age, joint pain, and muscle atrophy—each affecting the hind limbs—had statistically significant effects on canine physical activity (32).

Although OA has long been considered a joint disease, it involves neuro-sensitization and nociplastic pain mechanisms as CP progresses: it alters how the canine immune and nervous system processes pain by disrupting the balance between its inhibitory and facilitatory endogenous controls (25, 31). Consequently, compared to healthy dogs, those with OA may express somatosensory sensitization, experiencing maladaptive pain such as allodynia—pain perception from a normally non-painful stimulus—and hyperalgesia—an exaggerated pain response to a normally painful stimulus (31, 33, 34) In everyday life, the consequences of neuro-sensitization and nociplastic mechanisms may manifest in multiple ways within the human–dog dyad. Peripheral sensitization may result in mechanical or thermal hypersensitivity, expressed as allodynia or hyperalgesia, whereas central sensitization and nociplastic mechanisms may contribute to an increased sensitivity to normally innocuous sensory stimuli, such as touch or grooming (31). Clinically, these neurophysiological alterations may be reflected by avoidance behaviors, reduced social interactions, defensive aggression, agitation, vocalizations, or reluctance to participate in previously enjoyed activities.

As these behavioral changes emerge, the effects of OA-associated CP extend beyond the dog. Owners may progressively adapt their own behaviors and expectations, leading to modifications in daily routines, interactions, and the overall co-construction of the human–dog relationship. Thus, OA-associated CP is not solely an individual experience but a dyadic one, with reciprocal consequences for both partners.

Given the breadth of this disease’s canine impacts, it can be inferred that dyadic interactions are also altered (3, 35). Ideally, these repercussions should alert the owners who, recognizing that their dog is unwell, initiates a veterinary consultation – this marks the rupture point.

#### 2.1.2 Theoretical objectives and SDT

The theoretical objectives of dyadic deco-construction appear to be (11, 25, 30):

- Experiencing CP and its early signs through the progressive deterioration of canine, human, and dyadic well-being.
- For owners specifically:
- Doubting the normality of canine behaviors and daily dyadic routines.
- Recognizing that abnormality requires care.
- Deciding to seek professional veterinary assistance.

In light of SDT, deco-construction seems to reflect a gradual alteration of the canine, then human, and finally dyadic capacities for self-determination. The rupture point could correspond to an accumulation of frustrations in dyadic self-determination, which then becomes a motivational source prompting the owner to seek a veterinary consultation. In other words, the owner would reach a cognitive-behavioral threshold beyond which their concerns motivate them to take action.

### 2.2 Impacts of CP on canine self-determination

A study compared the well-being of *N* = 46 dogs suffering from CP (of musculoskeletal origin) with that of *N* = 143 healthy dogs (36). The Animal Welfare Assessment Grid (37) was used, consisting in four domains (36):

- *Physical health* (e.g., mobility, body condition, clinical assessment, eating and drinking).
- *Psychological health* (e.g., aggression toward the caregiver or strangers, frequent fear and anxiety, reaction to stressors).
- *Environmental* (e.g., choice, control, predictability, enrichment, and social factors).
- *Procedural* (e.g., behavior during assessment, daily routine changes, handling during assessment, and pain associated with the procedure).

Results suggested that, except for body condition, all these domains differed significantly between healthy and CP-affected dogs (36). In dogs with musculoskeletal CP, it was also observed (36):

- More frequent aggression.
- A positive and significant correlation between:
- Mobility and activity (*r_S_* = 0.91).
- Fear and anxiety frequency (*r_S_* = 0.88).
- That the severity of clinical signs increased with reduced mobility, as well as with the frequency of experience to frightening stimuli.
- That typical signs of musculoskeletal disorders such as gait changes, stiffness, lameness might manifest after behavioral changes such as increased fearfulness, prolonged recovery from a stressful event, reduced interest in social interactions, toys or play.

From the perspective of SDT, it is possible that dogs use aggression, at least partially, as a defensive and/or protective competence—that is, an explanatory competence that motivates the termination of an interaction (38). This behavior could allow them to regain a sense of control over the situation. However, canine aggression and frustration may deco-construct the way dyads previously managed their interactions, by introducing unpredictability.

In the specific case of canine OA, cognitive capacities and performance appear to be altered. In a study involving *N* = 20 OA dogs and *N* = 21 healthy dogs, it was found that (39):

- Spayed female OA dogs, but not males, showed impaired spatial memory in the object disappearance task compared with spayed healthy females.
- As the memory retention interval increased, OA dogs showed a steeper decline in spatial working memory performance than healthy dogs.

This performance decline can lead working dogs to early retirement. In a study involving *N* = 149 current or former police dog handlers and *N* = 182 dogs (40):

- *N* = 94 dogs were retired.
- The reason for retirement in *N* = 61 of them was their inability to meet the physical demands of the job.
- The main factor behind this incapacity in *N* = 42 dogs was degenerative musculoskeletal disease.

In terms of autonomy, compared to healthy dogs, those suffering from CP of musculoskeletal origin appear to have less choice, control, and predictability in their lives (36). They also engage less in enrichment activities (36). It is possible that enrichment poorly adapted to canine autonomous motivations and to the dog’s physical capacity to interact with it becomes a source of frustration, stress, and boredom (1, 41, 42). When dogs can no longer interact effectively with their environment, this affects not only their autonomy but also the owner’s and dyad’s competences during interaction—for instance, how can one lift a Saint Bernard into a car trunk if it refuses to cooperate?

Aspects related to attachment remain under-documented. It appears that dogs suffering from CP of musculoskeletal origin have poorer-quality social interactions and are more isolated than healthy dogs (10, 36). It is possible that, for these dogs, social interactions become associated with painful experiences. Consequently, they may learn that playing alone (to avoid undesired pain and maintain control of the interaction) is preferable to playing with another participant (36). Further research is needed on this topic.

Even before diagnosis is established, due to the early signs of OA, the canine, human, and dyadic competences required to fulfill self-determination appear to progressively deteriorate.

### 2.3 Rupture point: conjunction of canine and human argumentation

Rupture point was defined as a cognitive-behavioral threshold at which concern is translated into action. In a recent qualitative study, *N* = 32 interviews were conducted with *N* = 40 owners of *N* = 35 dogs with OA. The authors proposed a more nuanced interpretation of what is commonly referred to as the rupture point, dividing it into three phases (11):

- *Recognizing the problem*. Owners more easily identify acute changes—always associated with pain—than chronic ones, which are subtler and intermittent. However, in the context of canine OA, chronic changes, particularly subtile at the process beginning, are more frequent than acute ones. Canine factors such as breed, morphology, and coat type may also bias the owner’s accurate perception of the dog’s condition (43).
- *Considering the cause*. Owners’ perceptions appear to be influenced by the dog’s age, their own experience, and dyadic skills/education related or unrelated to OA. It is interesting to observe with different hypothetical acute pain scenarios to *N* = 367 dog owners (21), that their opinion to consult a veterinarian changed significantly with the educational intervention. Human sociodemographic factors are also associated with higher animal pain assessment scores, suggesting a potentially earlier recognition of symptoms (e.g., being female, being an experimented pet owner, having a higher personal income, being empathetic, and having a higher education level) (44).
- *Deciding what to do next*. Factors encouraging veterinary consultation included the dog exhibiting acute signs of discomfort, a strong human–dog bond, owners’ awareness of their own knowledge limitations, and trust in their veterinarian. However, owners’ inaction appeared to be reinforced by the subtlety of canine OA expressions, their belief that little could be done to manage the disease, fear of the veterinarian’s diagnosis, or concerns about future financial implications (11, 21). It is also possible that previous similar episodes of clinical signs in the dog reinforced the owners’ tendency to wait before seeking care (45). As previously indicated, stress-responses and behavioral changes such as increased fearfulness, prolonged recovery from a stressful event, a reduced interested in social interactions, toys or play occurred before mobility alterations in dog musculoskeletal CP (31). Owners only seeking veterinary attention when the presence of physical signs of disease are evident may result in a delayed veterinary attention (31). This is re-inforced by the activation of the inhibitory endogenous controls of pain (31), recently demonstrated as maintaining the OA dog (facing CP) comfort, and limiting the expression of functional alterations (31, 46).

From the perspective of SDT, there appears to be a discrepancy between the perceived competence and the real competence of owners to successfully navigate the rupture point. Various biopsychosocial, experiential, and environmental factors related to humans, dogs, dyads, and the pathology itself can shape the outcome of this process (11, 21, 43–45). In unfavorable cases, owners seem to develop an illusion of competence and control—that is, of autonomy—based on misinterpretations, lay beliefs, and stereotypes. This illusion leads them to underestimate their dog’s health condition, to deny the problem, and to adopt a passive or delayed stance, also called “the inaction paradigm” (11, 43, 47). This contributes to canine therapeutic wandering, undermines quality of life, and hinders the fulfillment of canine, human, and dyadic self-determination.

Belshaw et al. (11), noted that a strong human–dog bond could facilitate the rupture point. However, this claim was based on participants’ self-reports, and dyadic attachment was not directly measured. The study by Kogan et al. (21), cited previously, and the walking scenario provide additional insights. The predictors of the owner immediately contacting their veterinarian to schedule a consultation were (21):

- A higher score on the Lexington attachment to pets’ scale (LAPS).
- The fact that the owner:
- Felt very confident in their ability to detect their dog’s pain.
- Was very likely to consult a physician for themselves.
- Visited a veterinarian more than twice a year.

These findings suggest that the strength of a human’s attachment to their dog may accelerate the rupture point, but it interacts with other determinants, including how the person manages both their own health and that of their dog (21). Further research should examine reciprocal dyadic attachment in the context of OA-associated CP.

### 2.4 Practical feasibility of the theoretical objectives

In practice, the theoretical objectives associated with dyadic deco-construction appear only partially achievable. The impacts and progression of OA seem to lead to a dyadic deco-construction, marked by the degradation of canine, then human, and ultimately dyadic self-determination. Although this process is influenced by biopsychosocial, experiential, and environmental factors specific to the dogs, humans, dyads, and the pathology, owners often appear trapped in an illusion of competence, denial, and passivity. These attitudes delay the rupture point, as well as the establishment of a diagnosis and the dog’s access to appropriate treatment. Considering that the transmission and understanding of dyadic signals are facilitated by a more stable co-construction, it is possible that such stability also improves the overall deco-construction process (1). These conclusions should be interpreted with caution, as the concept of the rupture point and the effects of reciprocal attachment remain under-documented in the context of canine CP. Further research is needed to deepen these aspects.

Key takeaways:

- Dyadic deco-construction associated with CP related to canine OA first affects the dog and then, by a mirror effect, the owner and the dyad. It is influenced by biopsychosocial, experiential, and environmental factors specific to the dyad, its members, and the pathology.
- Owners’ interpretations of dyadic deco-construction often lead to denial and passive attitudes. This delays the rupture point and reinforces canine therapeutic wandering. Owner education dedicated to recognize signs of canine pain could reduce these attitudes and facilitate the dyad’s progression toward its rupture point.
- A more stable co-construction seems to enable dyads to navigate more effectively through the process of deco-construction that includes the rupture point.
- The rupture point and reciprocal dyadic attachment remain under-documented. More studies should be conducted on this topic.

## 3 Initiating the dyadic reco-construction

### 3.1 Overview of canine OA diagnosis and management

The ideal consultation for diagnosing canine OA consists of a thorough clinical history, including the owner’s responses to pre-consultation questions and illustrations of the dyadic daily life (5). This is followed by a complete physical examination (5), which includes checking vital signs and performing manipulation/palpation to detect pain, crepitus, joint effusion, thickening, or abnormal range of motion (5, 30). It is complemented by an orthopedic examination based on observations of the dog in motion and at rest, as well as radiographs of potentially afflicted joints to confirm the diagnosis (5).

After the diagnosis is confirmed, the triad—veterinarian, owner, and dog—discusses and selects therapeutic strategies for a personalized OA management plan. Typically, this first-line approach combines (5, 30):

- Weight reduction and nutritional management, including omega-3 polyunsaturated fatty acids (marine source).
- Regular and adapted physical exercise.
- Pharmacological options (e.g., nonsteroidal anti-inflammatory drugs [NSAIDs], anti-nerve growth factor [NGF] monoclonal antibody [mAb]). Depending of the diagnosed neuro-sensitization (increased input, functional or dysfunctional inhibitory endogenous controls) many neuro-pharmacological drugs are available.

### 3.2 Theoretical objectives and SDT

At this stage, in the context of this narrative review, dyadic reco-construction stems from the decision-making pivot integrated into the owner’s rupture point and is initiated by the veterinarian’s diagnosis. Its theoretical objectives primarily consist of enabling the triad to co-construct both the understanding of the disease and the plan for its therapeutic management (1, 11, 17, 35). From the perspective of SDT, this can be understood as a triadic attempt to clarify the dog’s health condition and to engage in discussion to establish an action plan. The action plan and the therapeutic strategies selected represent hopeful pathways that, once implemented, may allow the dyad to develop the skills needed to partially regain their capacities for self-determination.

### 3.3 Benefits and impacts

The delay in establishing the diagnosis is a critical variable. For dogs, the later the diagnosis is made, the more advanced the OA lesions are likely to be, thereby potentially limiting therapeutic possibilities – particularly in older dogs (47). Interestingly, as the nociplastic condition evolves, the inhibitory endogenous controls become over-solicited and a brain fatigue will occur, where the inhibitory endogenous controls are activated but dysfunctional (46). It happened that dogs with advanced OA presented a dual response to standard treatment (NSAID + Gabapentin) (46):

- Positive, for the OA dogs presenting maintained activated and functional inhibitory endogenous controls of pain.
- Negative when the inhibitory endogenous controls were dysfunctional.

In this specific study (46), it has to be noted that the authors feeling about the treatment efficiency being sensitive to the functionality of inhibitory endogenous controls of pain was mostly related to NSAID, and not gabapentin. Although gabapentin is increasingly used in veterinary prescriptions to relieve pain associated with canine OA, the scientific evidence supporting its efficacy in this context remains highly limited, quasi-inexistant. Further studies are needed to rigorously evaluate its clinical efficacy and its benefit–risk profile in the context of canine OA (48). *If no therapeutic option can be attempted or proves effective, this may ultimately undermine canine, then human, and finally dyadic self-determination*.

The impacts of the OA diagnosis on owners are ambivalent. A qualitative study conducted with *N* = 40 owners of *N* = 35 dogs with OA revealed that (17):

- If the dog was old at the time of diagnosis, most participants minimized the diagnosis and were not concerned about its impact on their lives. It could represent the logical conclusion of the inaction paradigm.
- If the dog was younger, the announcement of the diagnosis could trigger shock, sadness, or even guilt. Some owners expressed fear, confusion, and the feeling that the future would not unfold as they had imagined.

From the perspective of SDT, *owners’ lack of competence (and knowledge) or perceived competence regarding canine OA, as well as the dog’s age and the dyad’s lack of prior experience with OA, appear to influence owners’ motivation in two extreme ambivalent directions*:

- Those who struggle to take the diagnosis seriously and may become amotivated to properly follow veterinary recommendations.
- Those who dramatize the diagnosis and may develop attitudes of hypervigilance and hypercontrol toward their dog, thereby further impairing their own self-determination, as well as that of their dog and their dyad (16).

Having already experienced canine OA in the past seems to act as a protective filter: owners in this situation reported feeling more confident about the future and more autonomous (17).

### 3.4 Stress and canine self-determination

The experience of a veterinary consultation is stressful for dogs. In a study based on the reports of *N* = 906 dog owners, it appeared that (49):

- 7.4% of dogs showed signs of stress before leaving home.
- 39.7% expressed stress in the car.
- 52.9% expressed stress upon arrival at the clinic.

This stress is influenced by several factors (47, 49, 50):

- *Canine* (e.g., age, fear, and phobia).
- *Dyadic and triadic* (e.g., previous experiences of care received from the owner and/or the veterinary team).
- *Environmental* (e.g., familiarity with the place, type of handling, noise, and the presence of conspecifics or strangers).

Taken together, it appears that if dogs are not accustomed to being handled by their owners, often resist care attempts, and their owners react by reprimanding them, this increases the risk of aggression toward the veterinarian and their owners during visits (49). Furthermore, 46.2% of owners reported an increase in fearful and/or aggressive behavior from their dogs toward the veterinarian when the animal underwent a painful treatment (49).

From the perspective of SDT, the stress expressed by the dog during the consultation can be interpreted as a threat to its ability to satisfy its self-determination: dogs are compelled by humans to participate in a veterinary consultation. Despite the increasing use of cooperative care, if a dog has unmanaged CP and the veterinarian’s manipulations trigger pain, this could provoke a feeling of loss of control and undermine the dog’s autonomy during the interaction (50, 51).

Aspects concerning the impact of canine attachment during consultations are ambivalent and understudied. Based on small samples (*N* ≤ 33), several recent studies have analyzed veterinary consultations involving either dyads (52), or comparisons between dyads and dogs alone (53–55).

In the presence of their owner, dogs appear more willing to enter the consultation room (*p* < 0.05) and more relaxed at the beginning of the session (*p* < 0.01) (53). Several symptomatic measures of fear were lower when the owner was present than when absent (e.g., vocalizations, axillary temperature, and—only in females—heart rate) (55). However, whether the owner was present or not, reduced posture, avoidance, and escape behaviors did not differ (55).

During the consultation, it is possible that a dog’s visual or physical search for its owner represents an attempt to fulfill its fundamental need for attachment. The more stressed the dogs were, the more they sought visual contact with their owner (54). In Csoltova et al.’s (52) study, when owners were allowed to physically interact with their dogs, the dogs attempted significantly less often to jump off the examination table than when contact was not permitted (*p* = 0.002). However, whether contact was permitted or not, veterinary examinations induced a significant increase in lip-licking, heart rate, and maximal heart rate (52). In Helsly et al.’s (54) study, owners’ visual and verbal attempts to comfort their dogs had no significant effect on canine stress. Conversely, dogs’ anxiety signs intensified when owners displayed negative behaviors (54). These results suggest several key points. First, the owner’s presence, as well as visual or physical contact attempts, may subtly enhance canine well-being during consultations – though they are likely not the only contributing factors. Second, the consultation appears to push dyadic co-construction to its limits (1) and elicit canine emergency behaviors. *Thus, an unstable co-construction may further erode canine, then human, dyadic, and triadic self-determination throughout the veterinary consultation*.

### 3.5 Human self-determination

#### 3.5.1 Successful consultation

A systematic review (*N* = 17) indicated that several human factors influence the success of consultations for small animals presented to veterinarians for specific health problem (56):

- *Compliance* (e.g., clarity of recommendations, relationship-centered care approach, demonstrations of sympathy/empathy by the veterinarian, and the owners’ perception that the veterinarian spent enough time with them or did not rush the consultation).
- *Client satisfaction* (e.g., interactivity, warmth, and the veterinarian’s demonstrations of empathy).
- *Veterinarian satisfaction* (e.g., practitioner’s self-esteem, client cooperation, and the existence of a prior veterinarian–client relationship).

Although the literature remains limited and is constrained by methodological weaknesses, including small sample sizes and heterogeneous assessment tools (56), existing evidence indirectly identifies several conditions that may support the owner’s self-determination during the veterinary consultation. From an SDT perspective, the consultation should not only aim to address the dog’s health but also foster the owner’s fundamental psychological needs. Veterinarian and owner therefore share the responsibility of creating conditions that strengthen each other’s self-determination while working toward their common objective: improving the health and well-being of the animal.

Communication is central to this process, particularly when discussing emotionally challenging situations such as the diagnosis of a chronic disease or treatment failure (57). Regardless of the purpose of the consultation or the stage of the veterinarian–owner relationship, effective communication has been associated with better clinical outcomes, greater owner and veterinarian satisfaction, improved adherence to therapeutic recommendations, enhanced owner recall of information, and more efficient and accurate consultations (57–59). Conversely, poor communication may compromise clinical outcomes and even have health, safety, or legal consequences (57). Communication strategies such as active listening, open-ended questioning, empathic interactions, respectful language, support for owner autonomy, and a non-judgmental attitude contribute to building trust and facilitate information sharing, clinical reasoning, and therapeutic engagement (57).

Relationship-centered care, shared communication styles, and shared decision-making provide practical approaches for translating these principles into veterinary practice (60–64). By encouraging dialog and considering the owner’s preferences, values, experiences, and the broader human–dog dyadic context, these approaches help establish a collaborative partnership in which responsibility for long-term care is progressively shared. Although shared decision-making is recommended in veterinary medicine and valued by owners, its implementation remains inconsistent and is influenced by factors such as consultation duration, veterinarian experience, client characteristics, and appointment type (60, 62). More broadly, communication training remains insufficient within the veterinary profession, and the available evidence supporting best communication practices is still relatively limited (57).

From an SDT perspective, effective veterinarian–owner communication can be viewed as a key mechanism for supporting the owner’s psychological needs. Clear information and explanations strengthen competence by improving understanding of the disease and available treatment options. Respecting the owner’s values and involving them in decision-making nurtures autonomy, whereas trust, empathy, and active listening foster relatedness. Together, these processes are likely to promote engagement, adherence to therapeutic recommendations, and sustained participation in the management of OA-associated CP. Conversely, communication that frustrates these psychological needs may undermine motivation, weaken the veterinarian–owner partnership, delay recognition of the dyadic rupture point, and ultimately hinder dyadic reco-construction.

Finally, owner education constitutes an essential component of dyadic reco-construction from the time of OA diagnosis. Beyond the simple provision of information, it represents a progressive learning process through which, ideally, owners gradually acquire the knowledge and skills required to become active partners in the long-term management of their dog’s OA. This process may include (30, 65):

- Developing a better understanding of OA pathophysiology, therapeutic objectives, expected outcomes, and the rationale underlying multimodal management strategies.
- Acquiring the skills necessary to appropriately implement therapeutic recommendations, including medication administration, weight management, adaptation of physical activity, environmental modifications, and the performance of prescribed home-based physiotherapy or adapted exercise (35, 66, 67).
- Progressively developing the ability to evaluate treatment response, recognize clinically meaningful changes or adverse effects, and collaborate with the veterinarian in adjusting therapeutic strategies as the disease progresses.

From an SDT perspective, owner education primarily strengthens owners’ perceived competence, thereby promoting their sustained engagement in disease management.

#### 3.5.2 Key steps of the consultation

Regarding the standard consultation for OA diagnosis, a qualitative study involving *N* = 4 focus groups and a total of *N* = 26 veterinarians is particularly relevant (11). The results suggest that, to achieve their objectives, most veterinarians consider *clinical history* to be the most important part of the consultation (11), with the clinical examination serving only as a complement (11). However, clinical history may be influenced by:

- *Time constraints during consultations* (11, 47). Because of these limits, veterinarians may focus on the reasons for the owner’s visit and the health issues perceived and described by the owner, rather than fully exploring the dog’s health status (60).
- *Difficulties in information transfer between owner and veterinarian* (11, 60). The owner may fail to notice a problem with their dog and therefore not mention it to the veterinarian (11). Veterinarians may, in turn, struggle to extract relevant information from the owner’s discourse (e.g., differing priorities, misunderstandings of body language) (60).

The signs perceived during *clinical and orthopedic examinations* are influenced by several factors. First, practitioners’ sex, culture, and context appear to affect how veterinarians recognize and manage animal pain (68–71). Second, the lack of standardization, subjectivity, and variability in intra- and inter-observer data interpretation blur veterinarians’ perceptions and interpretations of manipulations and orthopedic signs (11, 25, 42). This may lead to a lack of confidence in the information gathered during the clinical and orthopedic examination. Finally, observations made during consultations represent a snapshot of the dog’s health status and the impact of a potential disease at a specific moment. However, this snapshot can be dysfunctional or incomplete (25). For instance, canine functional capacities vary daily, being influenced, among other factors, by the activities carried out beforehand (42) and the functionality of endogenous controls of pain (72).

*Radiographic* use is influenced by (11, 25, 73):

- Time allotted for the consultation.
- Canine age and clinical signs observed.
- Stage of OA.
- Technical constraints of the tool (e.g., positioning of the dog, contrast).
- Costs.

Its use is therefore not systematic in practice (11, 25). As radiography is the gold standard for confirming the diagnosis of canine OA, its absence may limit the provision of veterinary services truly appropriate to the dog’s health condition, potentially leading to misdiagnosis and inadequate management. In a recent longitudinal study of more than 3 years, radiographic OA progression in dogs has shown different trajectories: Non-progressors, slow-progressors and fast-progressors (74). Similarly to human OA, dogs could present an accelerated form of OA. Interestingly, the fast-progressors were more often affected at the stifle, and presented higher development of neuro-sensitization and functional impairment (72).

Afterward, the veterinarian informs the owner *of the findings, makes the diagnosis and proposes a treatment plan*. Therapeutic choices depend on several factors (5, 11, 30, 60, 75–77), even if they were mostly elaborated over surgical (acute) pain:

- *Clinical* (e.g., canine age, health status and comorbidities, therapeutic history, side effects, number and stage of affected joints, pain intensity).
- *Relational and contextual* (e.g., number of clients present during the consultation, owner’s skepticism toward the diagnosis, owner’s characteristics regarding pain management and analgesic use [e.g., cultural beliefs, age, gender, education, and profession, having or not undergone surgery themselves or with their animal], as well as the temperament/capacity of the dog, the owner, and the dyad to implement the veterinarian’s recommendations).

From the perspective of SDT, the limitations of the original co-construction, dyadic self-determination during the rupture point, and triadic self-determination during the consultation seem to influence the diagnostic process. The consultation can thus be analyzed as a zone of co-influence between human and canine capacities for self-determination. The fundamental psychological needs and motivations of the veterinarian, the owner, and the dog may either support or hinder one another, depending on the biopsychosocial, experiential, environmental, pathological, and technical factors unique to each (11, 25).

### 3.6 Practical feasibility of theoretical objectives

In practice, the theoretical objectives associated with the initiation of dyadic reco-construction appear to be only partially achievable. The consultation for diagnosing canine OA represents a pivotal stage where the dyad truly engages in understanding the situation and exploring potential solutions. The veterinarian acts as a mediator of meaning, catalyzing the co-construction of shared knowledge about the dog’s health status and the therapeutic strategies that can be implemented.

From the perspective of SDT, although this is never explicitly stated as such, the consultation appears to represent a space where the fundamental psychological needs and motivations of all triadic members can be simultaneously supported or hindered. The diagnostic consultation is therefore not merely a sequence of technical acts for medical validation but also a co-construction of understanding and planning future opportunities for dyadic self-determination fulfillment. However, not all triads experience this process in the same way. It is influenced by biopsychosocial, experiential, environmental, and technical factors specific to the dog, the human, the dyad, and the pathology.

Key takeaways:

- The diagnostic consultation represents the initiation of dyadic reco-construction.
- It appears to be co-constructed by all triadic members and influenced by biopsychosocial, experiential, environmental, pathological, and technical factors specific to each—along with the quality of the original dyadic co-construction.
- When the consultation supports the self-determination of each participant, it becomes a powerful lever for dyadic reco-construction by enabling a reliable diagnosis and an appropriate management plan.

## 4 Experiencing dyadic reco-construction through the implementation of care

### 4.1 Theoretical objectives related to SDT

The theoretical objectives of the next phase of the dyadic reco-construction process primarily involve experimenting with and adjusting, at home, the therapeutic strategies selected during the consultation to establish a new dyadic daily life. From the perspective of SDT, this stage is important because, progressively, it allows the members of the dyad to reco-construct and challenge their renewed capacities to satisfy their dyadic self-determination.

### 4.2 Benefits and impacts

Managing canine CP affects both the dyads and their members. Compared to owners of healthy pets, those whose companions suffer from chronic or terminal diseases appear to experience (78):

- A higher caregiver burden (*p* < 0.001).
- More symptoms of depression (*p* < 0.01), anxiety (*p* < 0.001), and stress (*p* < 0.001).
- A lower quality of life (*p* < 0.01).
- A greater caregiver burden associated with lower psychological and emotional well-being (*p* < 0.001).

However, a study based on SDT and involving *N* = 104 dog owners suggests that (79):

- Owners who provided psychological need support to their dogs experienced greater well-being, less psychological distress, and felt closer to their dogs than others. These effects were stronger than those of the support they received.
- On days when owners felt higher well-being (*p* = 0.012) and greater closeness (*p* < 0.001), they tended to provide more support to their dogs.

It appears that giving and receiving support are two distinct sources of satisfaction of human basic psychological needs (79). Although this study did not specifically address owners of dogs suffering from CP (and OA-associated CP), similar to the results of Dombestein et al. (8) regarding informal caregivers of human patients with long-term illnesses, it is possible that not all dog owners experience the negative impacts of their dog’s illness with the same intensity. *Perhaps some owners, whose intrinsic motivation lies in their dog’s well-being and who engage in a qualitative co-construction with their dog, develop protective competencies against this burden?* Unfortunately, this topic remains undocumented.

A recent study including 68 owners of dogs diagnosed with chronic kidney disease reported that only a minority of owners perceived their dog’s condition as having a negative impact on their professional activity (10%) or on their non-work-related activities (14%) (80). Furthermore, irrespective of the canine International Renal Interest Society (IRIS) stage, owners continued to perceive their dog as a source of emotional support and companionship (which could be reported as co-construction) (80).

These findings should, however, be interpreted with caution. Only 52% of the participants were employed, potentially limiting the interpretation of the reported occupational impact. In addition, most dogs (44/68) were classified as IRIS stages I–II, whereas only 20/68 had advanced disease (IRIS stages III–IV), and the disease stage was unavailable for four dogs. Finally, the treatment status of these 68 dogs was not reported. Notably, in the larger cohort from which this subgroup was drawn (*N* = 253), 54% of the dogs had never received treatment (80), making it difficult to determine the caregiving demands experienced by the owners included in this analysis.

Taken together, these findings suggest that the impact of a canine chronic disease on the owner is not uniformly negative and may depend on factors such as disease severity, treatment requirements, and caregiving demands. They also indicate that owners may continue to derive emotional support from their dog despite the presence of a chronic illness.

Specifically concerning canine OA, the owners’ quality of life—including their emotional well-being, physical functioning, daily activities, environmental adaptation, social functioning, rest, finances, and work—is affected (81). In other words, even after the diagnosis is made and therapeutic options are implemented, the disease continues to alter daily dyadic dynamics and human capacities to satisfy their self-determination. However, it remains unknown whether receiving a diagnosis of OA and being aware (and convinced) that therapeutic options exist, may have a protective effect on owners’ well-being and reco-construction.

For dogs, in addition to the direct effects of the disease (see Section 3), OA remains a progressive and incurable condition that may ultimately lead to euthanasia, including in cases where the disease is appropriately managed and welfare-oriented care is provided. A study involving *N* = 277 owners of dogs suffering from potential OA suggested that caregiver burden has significant indirect effects on the relationship between the clinical signs of the disease and the consideration of euthanasia (82). This relationship was significantly moderated by owner satisfaction (82). It appears that when owners perceive that:

- Canine OA has significant effects on the dog’s health, well-being—and, by extension, on canine, human, and dyadic self-determination.
- The proposed therapeutic options are too restrictive and not (sufficiently) effective; then euthanasia may be considered by both the owner and the veterinarian for humane reason.

### 4.3 Weight and nutritional management versus dyadic self-determination

This strategy is fundamental in the prevention and management of OA (30, 83). The causes of canine overweight and/or obesity are influenced by several factors (83–85):

- *Canine factors* (e.g., breed, health status, reproductive status, life stage, activity level – age and sex factors remain to be confirmed).
- *Human factors* (e.g., beliefs, values, choices, feeding methods and rules, exercise, living environment, age, body composition, education, and income).
- *Diet-related and management factors* (e.g., safety, relevance, frequency, quality, quantity, timing/method of feeding, and feeding environment).

From the perspective of SDT, dietary changes in a dog with OA can disrupt the dyad. Regarding dogs, it is possible that:

- The impacts of OA hinder the dog’s access to its food bowl—in other words, its autonomy, competence, and ability to act on its environment. However, dietary restrictions may lead to canine frustration (41).
- Due to the OA-associated CP experienced, they may diminish appetite (81).
- They may become demotivated by the new diet and refuse it (86).
- Dogs may develop behavioral problems that degrade dyadic interactions. Overweight dogs appear to exhibit more food-related problem behaviors (e.g., food guarding and/or stealing, reluctance to come when called, fear or reluctance to walk outdoors, and a higher likelihood of barking/growling/snapping at other dogs or strangers) (87). The causal relationship between these behaviors and obesity has not been established (87).

These observations suggest that, in cases of canine weight and nutritional management, the entire feeding experience should be *adapted to the dog’s basic psychological and physiological needs and to the most intrinsic sources of its motivation*.

Owners’ self-determination capacities also appear to be challenged in contexts of canine overweight or dietary changes. Many owners lack competence and autonomy to:

- Recognize and correctly assess canine overweight (84)—they tend to underestimate it (85, 88).
- Properly measure/weigh food portions and accurately assess the dog’s activity level (83).
- Follow veterinary recommendations (89).

Owners may anthropomorphize their dogs (87) and believe that giving treats strengthens their dyadic relationship or maintains the dog’s happiness (18–20). It is worth recalling that adult human attachment can be divided into several styles (22, 23):

- Anxious attachment, characterized by concern about the availability and reliability of attachment figures.
- Avoidant attachment, referring to a tendency toward distrust and emotional distancing from attachment figures.
- Secure attachment, in contrast to insecure attachment (anxious or avoidant), is based on the belief that attachment figures will be available and responsive to one’s needs.

In the study by Coy et al. (19):

- Greater attachment anxiety predicted greater concern related to the evaluation of the animal, which in turn predicted greater attention toward the animal.
- Higher avoidant attachment was associated with less pet-related evaluation concern and less care and attention toward the pet.

Whether attachment was anxious or avoidant, the level of caregiving appeared to depend on pet-related rejection sensitivity, particularly owners’ concerns that their pet might judge them negatively or hold a poor opinion of them (19). It is possible that individuals with anxious attachment give more treats to their pets than others, attempting to ensure that their animals evaluate them positively (19, 20). These conclusions should be interpreted with caution, however, as Coy et al. (19), did not measure attachment with a dyad-specific tool.

### 4.4 Regular/adapted exercise and environmental modification versus dyadic self-determination

To address this strategy, dyadic walks appear ideal as they are accessible to most owners. A qualitative study involving *N* = 40 owners of *N* = 35 dogs with OA explored dyadic adjustments made to walks following the disease diagnosis (35).

From the perspective of SDT, on the canine side, the signals sent can be interpreted as expressions of discomfort or as attempts to negotiate the evolution of the interaction (e.g., slowing down, stopping more frequently, spending more time sniffing, sitting and/or looking at the owner when facing an obstacle or after walking a certain distance or duration) (35). These expressions seem to reflect dogs’ desire to regain or maintain control over the interaction, despite pain, to preserve their competence and autonomy. Looking at the human may be interpreted as a request for emotional support within an attachment-based relationship (35).

Owners are not insensitive to canine cues (35). Depending on their interpretations, they may (35):

- Before engaging in the activity: assess the dog’s gait and attitude to decide the structure of the walk (e.g., length, speed, duration, location, type and intensity of activity).
- During the walk: modify it actively to protect the dog (e.g., shorten or slow down walks).

However, owners differ in their evaluations. For example, being female, being a pet owner, having a higher personal annual income, and higher education levels have been significantly associated with higher animal pain assessment scores (44). It is possible that these factors also influence the interpretation of CP expressions during reco-construction. Finally, although walk adjustments may generate frustration (35), the strength of owners’ attachment to their dog seems, to some extent, to protect their continued investment in caregiving (17). This suggests that human attachment and motivation to care for the dog may be sufficient to sustain caregiving behaviors (35).

In addition, owner–dog dyads may have access to complementary and alternative interventions (e.g., physiotherapy, acupuncture, aromatherapy) as well as environmental modifications (48). Although evidence supporting their biomedical efficacy for alleviating OA-associated CP remains limited or inconsistent (48), these interventions may nonetheless provide relational and functional benefits when considered through the lens of SDT.

Environmental modifications are generally tailored to the individual dog rather than implemented according to standardized protocols, and their design largely depends on the experience of both the veterinarian and the owner (90). When integrated into the dyad’s daily routines, such adaptations may promote autonomy, competence, and functional comfort for both the dog and the owner, while strengthening the quality of their interactions (see Section 2.2). Similarly, home-based exercises, including simple physiotherapy programs, require owner training, knowledge acquisition, and sustained, progressive engagement (90). Although empirical evidence in canine OA-associated CP remains scarce, findings from *human medicine* suggest that:

- Owners who are intrinsically motivated to engage in these interventions, and who successfully internalize them as part of their caregiving role, may develop a stronger sense of competence and agency in supporting their dog’s health and well-being (8, 90).
- Active participation in these interventions has the potential to reinforce both the relational (attachment) and functional dimensions of the owner–dog partnership (91).

The successful implementation of complementary and alternative therapies depends on multiple interacting factors, including the type of intervention, the quality of the initial dyadic co-construction, the dog’s individual needs, and the owner’s availability, preferences, and caregiving abilities (1, 90). Conversely, when these interventions are perceived as ineffective, excessively demanding, or financially burdensome, they may frustrate the owner’s psychological needs for autonomy and competence, thereby contributing to caregiver burden and ultimately altering long-term engagement (see Sections 4.2. and 4.5) (8).

### 4.5 Medication administration versus dyadic self-determination

The effectiveness of pharmacological strategies depends, among other factors, on dyadic therapeutic compliance. It is influenced by several factors related to (30, 66, 67):

- *The medication* (e.g., physical/social risks, side effects, dosing schedule, and method of administration).
- *The dog* (e.g., age, health status, and comorbidities).
- *The owner* (e.g., sex, perceived discomfort from the dog’s symptoms).
- *The veterinary consultation* (e.g., discussion of dosage regimen in relation to dyadic routine, duration of the consultation, timing of the consultation and treatment).

These factors can influence the satisfaction of dyadic self-determination. If medication administration is associated with aversive training, use of physical restraint, or lack of anticipation of potentially painful interactions, this may increase canine frustration (1, 49, 51). It may hinder the dog’s competence, engagement, and cooperation, as well as its motivation and willingness to receive medication. Conversely, canine motivation can be enhanced when:

- Cooperative care methods are employed (51).
- The owner and veterinarian collaborate to select a palatable medication with a practical form of use and ingestion (66).

For owners, managing CP pharmacologically represents a major burden. In the context of OA, recent studies suggest that:

- Owners often struggle to feel competent and autonomous when facing the vast array of veterinary therapeutic recommendations and the associated financial constraints (16, 17).
- Their attachment to their dog exacerbates these tensions: some feel they have failed their dog when no effective medication is found, while others persist in a continuous quest for a solution to restore their dog to normal (17).

Owner satisfaction with the treatment received is crucial for therapeutic adherence. Factors influencing this satisfaction include treatment efficacy, mode and frequency of administration, ease of integration into daily routines, and the range of available therapeutic options (81). In Belshaw et al. (17), most OA dogs received a prescribed treatment (NSAIDs), and nearly all owners reported a perceived decrease in efficacy over time as the disease progressed. This could also be related to the common occurrence of gastrointestinal, hepatic or renal side effects, associated with NSAID. They also invested in numerous tools, remedies, and over-the-counter products but were often disappointed—the results did not meet their expectations in terms of both concept and effectiveness (17). Perceived loss of control over the disease’s progression, combined with owners’ emotional responses to their dog’s clinical signs, are significant predictors of caregiver burden (92). It is interesting, in such context, to highlight the efforts of the pharmaceutical industry to provide long-term treatment requiring a reduced number of manipulations and administration: this was the case with mavacoxib, available in European Union, with a monthly administration over a maximum of 6.5 continuous months; the arrival of anti-NGF mAb in the OA veterinary market revolutionized the therapeutic approach: monthly subcutaneous administration of bedinvetmab was recently followed by three-months product, izenivetmab.

### 4.6 Long term monitoring

Canine OA is a progressive chronic disease that requires long-term management and regular reassessment of clinical signs, treatment efficacy, adverse effects, and quality of life (30, 65). From an SDT perspective, long-term monitoring extends beyond evaluating disease progression. It represents an ongoing relational process through which the veterinarian, owner, and dog continue to support the dyadic reco-construction initiated following diagnosis. Sustaining this process requires the long-term engagement of all three partners as the disease evolves.

Regular follow-up consultations provide opportunities to adapt therapeutic plans according to disease progression, changes in functional abilities, owner expectations, and the evolving needs of the human–dog dyad (30, 65). They also allow veterinarians to identify emerging concerns, address misconceptions, reinforce realistic expectations regarding the progressive nature of OA, and recognize early signs of renewed dyadic disruption before they culminate in another rupture point.

Supporting the owner’s fundamental psychological needs is likely to enhance long-term engagement in disease management. Educational interventions, guidance, and therapeutic adjustments strengthen competence by improving understanding of OA and its management. Shared decision-making and individualized treatment planning foster autonomy by encouraging active participation in therapeutic choices, while a trusting and collaborative relationship with the veterinarian supports relatedness. By sustaining these three psychological needs over time, long-term monitoring may promote continued adherence to treatment, facilitate repeated cycles of dyadic adaptation, and contribute to maintaining both quality of life and resilient human–dog relationships throughout the progression of OA.

### 4.7 Practical feasibility of theoretical objectives

The theoretical objectives of dyadic reco-construction appear to be partially achievable. From the perspective of SDT, feeding, walking, and medication administration represent moments where the quality of dyadic reco-construction is expressed. Although rarely conceptualized this way, it seems that a dyad with a strong capacity to satisfy its self-determination and a qualitative co-construction can transform the constraint of therapeutic strategies into an act of collaborative care, while a weakened dyad risks turning it into a source of tension. At home, the success of ongoing management depends, among other factors, on supporting the fundamental psychological needs and motivations of both dyadic members. When analyzing reco-construction, it becomes evident that canine self-determination in this context is relative. The human appears to act as the guarantor of canine self-determination, preserving the canine, human, and dyadic daily life from the future impacts of OA.

Key takeaways:

- The at-home adaptation of the therapeutic management discussed during the consultation represents the practical implementation of dyadic reco-construction.
- It is influenced by biopsychosocial, experiential, and environmental factors related to the owners, the dogs, and the pathology, as well as by the quality of the original co-construction.
- It illustrates individual and collective attempts to regain dyadic self-determination. However, humans remain its ultimate guarantors.
- Aspects related to human, canine, and dyadic attachment are underexplored, and further studies should be conducted on this topic.

## 5 Supporting dyads in anticipating the rupture point and navigating the deco-/reco-construction more effectively

### 5.1 Education and prevention

The rupture point should not result from an accumulation of shortcomings and misunderstandings. It should instead represent the outcome of a shared understanding process, supporting a transition toward dyadic reco-construction. The responsibility for the owner’s lack of self-determination in recognizing the early signs of canine OA cannot rest solely on them. In a recent study involving *N* = 738 dog owners, participants reported (93):

- Being more familiar with the importance of mental engagement, daily quality-of-life assessment of their dog, vaccinations, and parasite prevention than with breed-related diseases (*N* = 307) or care for senior animals (*N* = 276). Yet, they expressed interest in learning more about these topics (*N* = 287 and *N* = 276, respectively).
- Feeling that knowing more about their dog’s primary healthcare reduced their overall anxiety about their pet’s health (*N* = 634) and helped them feel more in control (*N* = 665).
- Considering preventative care as a good investment in their dog’s long-term health (*N* = 691), allowing them to prevent future pain (*N* = 687) and serious health issues (*N* = 683).

It is possible that:

- Veterinarians focus their preventive discourse more on emergency management than on helping dyads anticipate, detect, assist in diagnosis, and manage future CP.
- Owners receive the information (6, 30, 65, 83) but struggle to process it, connect current behaviors with potential future consequences, and generate autonomous motivation to act now to prevent later repercussions.
- Societal regulators (e.g., city, state/province, or country) influence the responsibilities of pet owners by defining their obligations. In France, the Certificate of Commitment and Knowledge (94), which is mandatory for a new owner and signed with the person transferring (selling) the animal, highlights (95):
- The owner’s individual responsibility and long-term commitment, supporting a form of decision-making autonomy framed within a sense of duty.
- The general dyadic needs for competence and attachment. However, it does not focus on breed-specific characteristics.
- Such a mechanism could also prepare owners for the possibility of chronic degenerative diseases such as OA, notably through standardized and regularly updated appendices specifying needs and warning signs (e.g., physical, behavioral, and health-related characteristics) for each breed and for mixed-breed dogs. This could help lay the foundations for stronger dyadic self-determination, by preparing adoption and co-construction (1, 93), and triadic self-determination, by supporting CP prevention and the processes of deco-construction and reco-construction. This hypothesis would need to be tested in a close future.

### 5.2 Checklist and screening tools

These tools are not intended to diagnose OA-associated CP. Their purpose was to shed light on indicators that manifest in daily dyadic life and that should prompt veterinary investigation to potentially diagnose a condition. Regarding canine OA, a recent study proposed a checklist (96). The advantages of this list are that the questions are:

- Short, simple, and limited in number.
- Focused on functional abilities and canine performance.
- Some specifically address dysfunctions that affect dyadic interactions, which increases the likelihood that owners will recognize potential signs.
- They could help trigger the owner’s rupture point earlier and initiate reco-construction.

The disadvantages are:

- The subjectivity of the answers provided.
- Since owners often struggle to identify CP in their animals (11, 16, 17) they may inaccurately complete certain questions.
- Co-construction and daily dyadic life were not documented (1): if the dog is constantly tethered outside or never has the opportunity to climb stairs, the owner may be unable to answer several of the proposed questions.
- The questionnaire was under construction and its validation, at that time, was deficient. An OA diagnosis was confirmed by a veterinarian in 38% (188 of 500) of dogs. Balance of sensitivity and specificity across the original group of nine screening questions was optimized to approximately 88 and 71%, respectively, after elimination of three questions (96). Further validation is needed.

To improve this checklist, it should be designed to document dyadic co-construction, to assess the dyad’s and its members’ capacity to acquire self-determination throughout their interactions, and to maintain it over time.

Preventive approaches should encourage both veterinarians and owners to recognize the importance of human–dog dyadic co-construction and to understand how it may be progressively altered by the onset and evolution of diseases such as OA. Greater awareness of these relational changes could facilitate earlier recognition of subtle behavioral modifications, prompt timely discussions during veterinary consultations, and ultimately support earlier intervention before dyadic disruption reaches a rupture point.

To support this preventive perspective, future assessment tools could incorporate questions documenting changes in dyadic routines and interactions rather than focusing exclusively on isolated canine functional impairments. For example, questionnaires could explore changes in shared activities (e.g., walks, play, climbing stairs, jumping into the car, proximity during rest, or social engagement), the frequency of adaptations initiated by the owner, and the owner’s perception of the dog’s willingness or motivation to engage in these interactions (Table 1). Longitudinal questions comparing the dog’s current behavior with its own previous baseline could further improve the detection of subtle changes associated with CP and the early stages of dyadic deco-construction. The increasing availability of digital tools is also expected to facilitate such longitudinal monitoring by enabling, for example, comparisons of videos documenting changes in posture, gait and shared activities over time, therefore providing a richer understanding of the progressive evolution of the human–dog dyad.

Similarly, screening tools could move beyond identifying established disease by incorporating indicators that reflect the owner’s perception of emerging changes within the dyad, together with their motivation and intention to seek veterinary advice. Such measures could help identify owners who recognize early alterations in the human–dog relationship and encourage preventive intervention before more profound dyadic disruption occurs.

### 5.3 Activity monitors

Accelerometer-based activity sensors are widely used to monitor locomotor activity in dogs are increasingly recognized as valuable tools for assessing animal comfort, usual mobility patterns, health status, and pain (25, 46, 97–103). However, their use, reliability, and comparability across studies can be influenced by multiple factors, including:

- *The device characteristics* (e.g., sensory type, sampling frequency, memory capacity, batteries life, device placement, attachment method, risk of device loss or detachment).
- *The animal-related factors* (e.g., species, age, body weight and conformation, living alone or in group, health status, and concomitant medication use).
- *The environmental variables* (e.g., accessibility and connectivity, control of housing conditions, exterior temperature fluctuations, standardization of daily routines, human interaction).
- *The data* (e.g., big datasets generation that require cloud-based downloading, extensive processing to simplify and structure raw outputs, identification and extraction of relevant day- or period-specific data, time-consuming analyses, and appropriate visualization methods to ensure meaningful interpretation).

Although numerous activity-monitoring devices (also referred as actimeters) are commercially available, only a limited number have undergone rigorous evaluation or validation studies (46, 98–110). For canine use, actimeters are typically attached to the collar on the ventral side of the neck, a placement that is practical, well tolerated, and provides reliable measurements of locomotor activity (103). In the context of the topics addressed in this review, actimeters offer several key advantages:

- They objectively measure the animal’s activity while preserving the dog’s competence and autonomy in its interactions with its owner and natural environment.
- If abnormal activity patterns are detected, they may contribute to the early identification of functional alterations associated with OA-associated CP. From a longitudinal perspective, analyzing deviations from each individual’s previous (baseline) activity levels could help detect subtle changes preceding the owner’s conscious perception of abnormality. These early signals may help anticipate the owner’s rupture point and potentially optimize therapeutic management.
- When used daily by owners as monitoring tools, they may also facilitate communication with the veterinary team, which will need to develop greater expertise in actimetry.

The impacts of OA-associated CP on canine activity are not always straightforward to interpret, as factors such as age or owner-induced changes in activity may also influence behavior (103). Consequently, the processing, analysis, and interpretation of actimetry data remain complex and may require additional validation (98–102, 104–109). Moreover, these devices primarily measure the quantity of locomotor activity and do not directly capture the relational dimension of interactions between the dog and its owner. This lack of consideration of dyadic co-construction may limit the interpretation of behavioral anomalies and the early detection of deco-construction processes preceding the rupture point.

Several studies have also shown that actimetry can complement the results of podobarometric gait analysis by capturing weekly activity under free-living conditions, which may better reflect the dog’s day-to-day functional comfort (100–102, 106, 107). However, the analysis of large actimetry datasets remains challenging. Previous studies in OA dogs have often reported limited treatment responsiveness and weak concurrent validity with other outcomes (111–114). Despite these limitations, several studies have detected significant treatment responses (97, 113, 115, 116), and some have reported concurrent validity with podobarometric gait analysis and clinical metrology instruments (98–100, 109).

Notably, the validity of actimetry has been strongly supported in studies involving nutraceutical interventions (100, 101) and green-lipped mussel-based omega-3 supplementation (97, 108). More recently, decreased mobility measured by actimetry in canine OA-associated CP has been associated with increased neuro-sensitization and altered endogenous inhibitory control function (46).

Consistent with the recognized behavioral signs of canine OA, such as reduced activity, decreased mobility after exercise, or stiffness after rest, actimetry studies have reported consistent patterns: OA dogs receiving placebo typically show no meaningful improvement in activity intensity and often exhibit a gradual decline over time (25, 46, 98–102, 104–109). Together, these findings support the potential development of algorithms capable of interpreting longitudinal actimetry patterns in dogs.

Concurrent validation studies should evaluate quantitative measures of canine locomotor activity alongside the contextual and relational dimensions of daily human–dog interactions. For example, combining actimetry with owner-reported diaries, geolocation during walks, environmental data, and artificial intelligence algorithms capable of detecting changes in dyadic interaction patterns could provide a more comprehensive interpretation of fluctuations in activity. Such multimodal approaches may help distinguish reductions in activity attributable to OA-associated CP from those resulting from environmental influences or owner-driven adaptations.

Longitudinal analyses focusing on individualized dyadic routines and deviations from previously co-constructed interaction patterns could facilitate earlier detection of abnormalities associated with OA-associated CP and improve anticipation of the dyadic rupture point. They may also provide objective indicators of disease progression, including changes consistent with increasing neuro-sensitization and its deleterious consequences, as recently reported in canine OA (46). Finally, these approaches could test the hypothesis that measures of human–dog relationship quality and dyadic functioning predict veterinary help-seeking behavior more accurately than locomotor actimetry or mobility scores alone.

### 5.4 Recommendations

Here are some suggested improvements to tools that could help dyads navigate deco−/reco-construction more effectively and anticipate the rupture point in the context of canine OA-associated CP:

- Strengthen education and prevention: owners often struggle to connect present dyadic behaviors with their potential future consequences. Better owner awareness before adopting an animal could foster more autonomous motivation toward both a high-quality co-construction and the prevention, detection, diagnosis, and management of canine OA-associated CP.
- Checklists are useful for identifying the owner’s rupture point but remain limited by the subjectivity of responses, and the standardization of the checklist does not address the variability inherent to individualized modifications.
- Further studies should validate the ability of activity monitors (looking more fiable than mobility scores) to detect the change in the level of activity which may be affected in dogs suffering from CP in OA.
- Both checklists and activity monitors should incorporate measurements of dyadic co-construction normality to better highlight emerging abnormalities potentially related to OA-associated CP.
- Determination of neuro-sensitization could represent an attractive avenue. Integration of artificial intelligence could be a major catalyzer in developing and validating efficient and practical tools.

### 5.5 Limitations

This narrative review presents several limitations that should be acknowledged. First, the proposed framework remains conceptual and exploratory, as the application of SDT to canine OA-associated CP and human–dog dyadic relationships has not yet been empirically validated (1). Although SDT is well established in human healthcare context, its application to dogs raises important theoretical and methodological challenges, particularly regarding the interpretation of canine self-determination capacities. Therefore, some of the interpretations proposed in this review remain hypothetical and are based on behavioral observations and human interpretation of canine responses.

Moreover, the concepts of dyadic co-construction, deco-construction, reco-construction, and rupture point have not yet been operationalized using validated assessment tools (1). This manuscript should thus be viewed as a narrative and conceptual contribution intended to generate hypotheses and encourage future research, rather than as a source of clinical or experimental validation.

In addition, several dimensions discussed in this review, particularly reciprocal attachment, dyadic interactions, and the influence of biopsychosocial and environmental factors, remain insufficiently explored in veterinary medicine and rely on a limited body of literature. Finally, as a narrative review, this work does not follow the methodological framework of a systematic review or meta-analysis and may therefore be influenced by selection and interpretation biases in the literature considered.

## 6 Conclusion

In line with SDT and the notion of dyadic co-construction (1), deco-/reco-construction in the context of canine OA appear as complex, adaptive, and progressive processes involving humans (owners and veterinarians), dogs, and the pathology itself.

Deco-construction manifests as a gradual decline in the satisfaction of canine self-determination, which, by a mirror effect, extends to the human and dyadic levels. These deteriorations may lead the owner to their rupture point, prompting a veterinary consultation. However, this process is often hindered by the subtlety of the early signs of canine OA-associated CP, as well as by owners’ illusion of competence, denial, or passive attitudes promoting the inaction paradigm (11, 21, 45).

Reco-construction can occur with two steps. First, as a dyadic attempt to make sense of the dog’s health condition and to search for potential solutions. The diagnosis and therapeutic strategies are then co-constructed by the members of the triad, ideally respecting each one’s capacity for self-determination. Second, at home, these therapeutic strategies are tested, adjusted, and then either integrated into or rejected from the dyadic daily life. When effective, they represent—at least temporarily—the reestablishment of canine, human, and ultimately dyadic self-determination. However, the co-construction of a new canine self-determination remains conditional, fragile, and largely dependent on the owner’s engagement and interpretation of the dog’s condition. If the owner does not seek veterinary care, declines the diagnosis, or refuses the therapeutic strategies proposed by the veterinarian, the dog may remain caught in a state of therapeutic wandering. Consequently, the transition toward a new therapeutic trajectory and the associated dyadic reco-construction do not rely solely on the owner’s initiative. Because many dogs routinely attend veterinary appointments for vaccination or preventive healthcare, these encounters also provide valuable opportunities for veterinarians to detect early clinical signs consistent with OA and initiate timely intervention. Not all dyads possess the same resources to navigate these processes. Deco-/reco-construction and the occurrence of the rupture point depend on biopsychosocial, experiential, environmental, and technical factors specific to the dog, the human, and the pathology. Nevertheless, a more stable initial co-construction (1) appears to offer a better starting point for navigating these processes.

From a methodological perspective, facilitating an earlier rupture point recognition by owners and a more effective passage through deco-/reco-construction relies on a toolkit of complementary instruments. These range from owner education and prevention to the use of checklists and activity monitors (as well as other sensorial tools). Their primary goal is to strengthen owners’ competence and autonomy throughout these processes and to foster better triadic collaboration. However, despite owners’ interest in learning more about their dog’s future health (93), they still struggle to transform their knowledge into autonomous motivation and sustained behavioral change. Educating owners about quality co-construction and the processes of deco-/reco-construction would be most beneficial before the actual adoption of an animal. While checklists and connected devices are promising tools (96, 103), they should better integrate the specificities of co-construction and daily dyadic life to enable earlier detection of anomalies related to OA-associated CP. These tools appear particularly important since many owners still interpret the early signs of OA as expressions of normal aging (35).

Conceptualizing OA-associated CP through the lens of SDT offers a promising pathway to anticipate deco-construction, support reco-construction, and initiate the owner’s rupture point. However, animal OA-associated CP has never been analyzed in relation to SDT, nor defined as a process of deco-/reco-construction. Dimensions related to competence and autonomy—canine, human, and dyadic—are often explored, yet attachment remains largely neglected. Nevertheless, reciprocal dyadic attachment could represent a key explanatory mechanism underlying all the processes discussed (18–23). Further research is needed to address these gaps.
